# Case Report: Minimally invasive repair of a traumatic abdominal wall hernia in a child with a fascial closure device

**DOI:** 10.3389/fsurg.2024.1391533

**Published:** 2024-07-05

**Authors:** Huiyu Lu, Zemin Zhang, Jun Chao, Chuanguang Zhang, Guoqing Zhang, Shujie Tao, Qingtao Yan

**Affiliations:** ^1^ School of Clinical Medicine, Shandong Second Medical University, Weifang, China; ^2^ Department of Pediatric Surgery, Weifang People's Hospital, Weifang, China; ^3^ Department of Dermatology, Weifang People's Hospital, Weifang, China

**Keywords:** traumatic abdominal wall hernia (TAWH), pediatric trauma, laparoscope, fascial closure device, abdominal wall hernia suture repair

## Abstract

Abdominal trauma is common in daily life, but a traumatic abdominal wall hernia (TAWH) in children is rare. A TAWH is caused by a huge external force that leads to subcutaneous muscle and fascia rupture, while the skin remains intact. As abdominal pressure increases, the abdominal contents protrude, forming a lump. A TAWH is highly susceptible to missed diagnosis because of other severe injuries. We report a case of a 2-year-old boy with a TAWH who developed a prominent subcutaneous mass on the right side of his abdomen after abdominal trauma; the size of the mass changed significantly with abdominal pressure and crying. In this case, we used a new approach of laparoscopic suture repair technique with the assistance of a fascial closure device and achieved good results. We found that this method offers the advantages of minimally invasive surgery, fast recovery, and no visible surgical incisions. There was no recurrence after 8 months of follow-up.

## Introduction

A traumatic abdominal wall hernia (TAWH) is a protrusion of abdominal organs or tissues into the body surface due to the rupture of muscle tissue and fascia associated with blunt trauma; it occurs without skin penetration and without signs of a hernia defect at the site of injury (1). Among children, a “handlebar hernia” caused by bicycles is the most common (2). In the past, we often used open or laparoscopic repair techniques. However, in this case, we successfully treated a child with a TAWH by laparoscopy with the assistance of a fascial closure device.

## Case description

**Figure 1:**
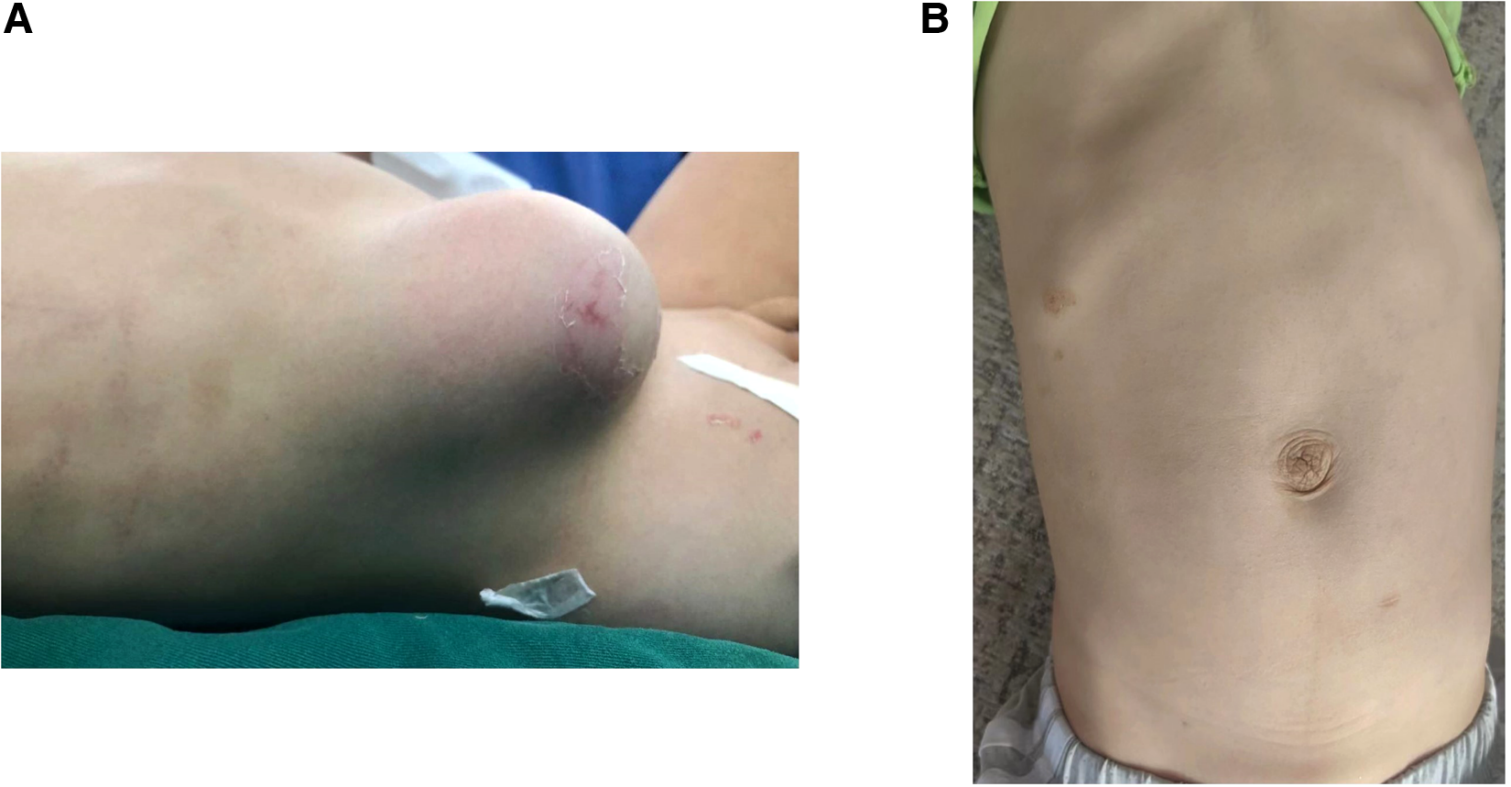
(**A**) Protruding mass seen in the right abdomen after the injury. (**B)** Abdominal condition of the patient followed up 8 months after surgery.

**Figure 2:**
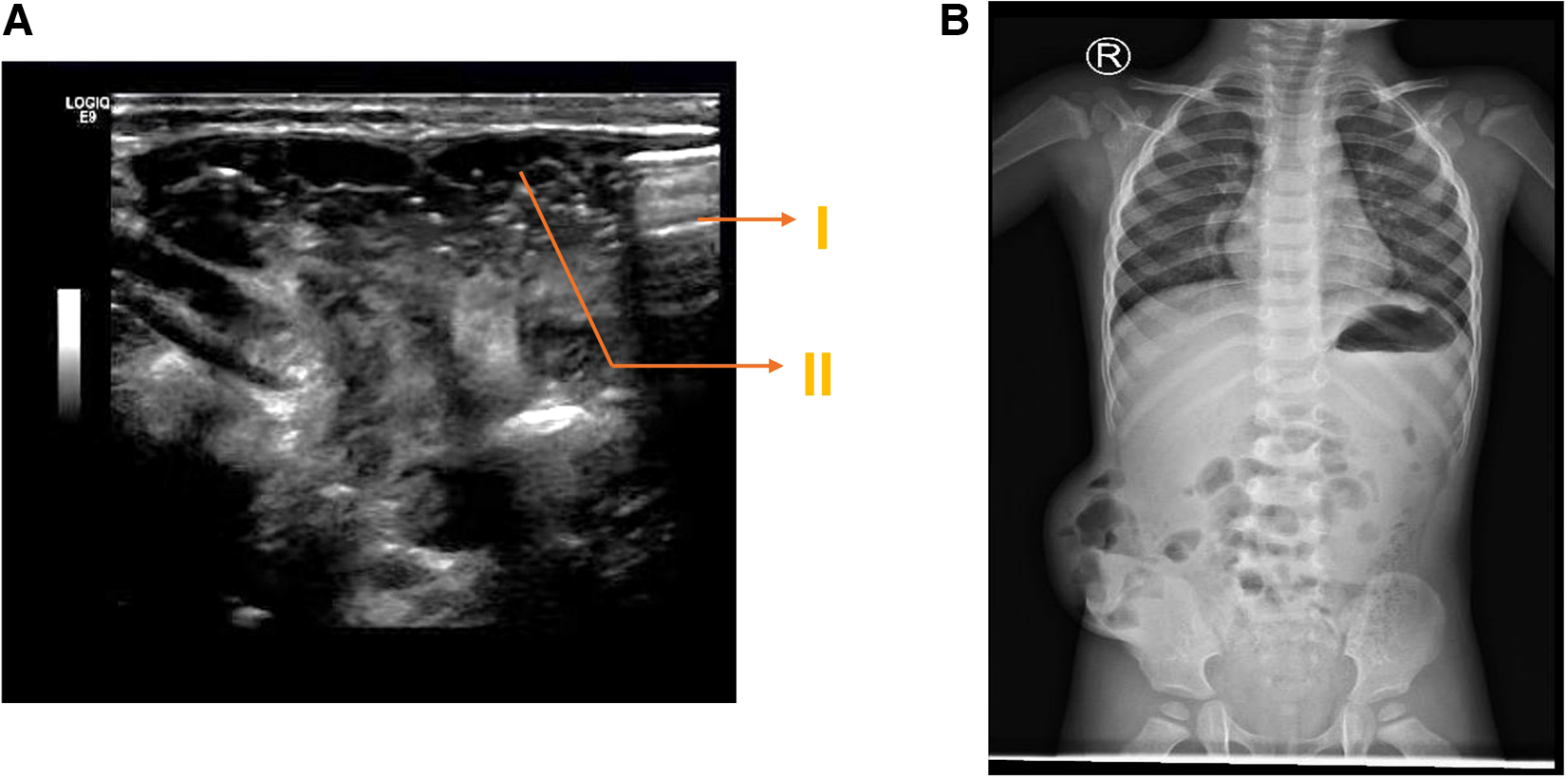
(**A**) Abdominal ultrasound prompts TAWH before operation. (I: muscle and peritoneal layer rupture caused by trauma; II: intestinal contents in the abdominal cavity protrude subcutaneously through muscle and peritoneal rupture). (**B)** Abdominal x-ray film before surgery.

A 2-year-old boy was injured in a car accident and developed a subcutaneous mass on the right abdominal wall that persisted for 4 h. No special treatment was administered outside the hospital. The patient had been in good physical health and had no prior illnesses. Physical examination revealed the following: body temperature: 36.5℃, pulse: 76 beats per minute, blood pressure: 94/64 mmHg, body weight: 11 kg. The protrusion was on the right abdomen, and the palpable subcutaneous mass measured about 8 × 6 × 2 cm in size, was soft, and able to retract. It increased with the crying of the child and decreased when the child was quiet, accompanied by local tenderness. Gut sounds were audible at the mass, but it was negative for mobile voiced sounds (Figure 1A). Ultrasound examination showed mixed echoes detected at the right abdominal protrusion, ranging about 7.1 cm × 5.0 cm × 1.1 cm, with clear boundaries and connection to the abdominal cavity, suggesting an abdominal wall hernia (Figure 2A). An abdominal x-ray film showed protrusion of the right portion of the intestinal tract from the right lower abdominal wall (Figure 2B). Therefore, the child was diagnosed with a traumatic abdominal wall hernia. After admission, we planned to perform laparoscopic surgery to repair the abdominal wall hernia.

**Figure 3:**
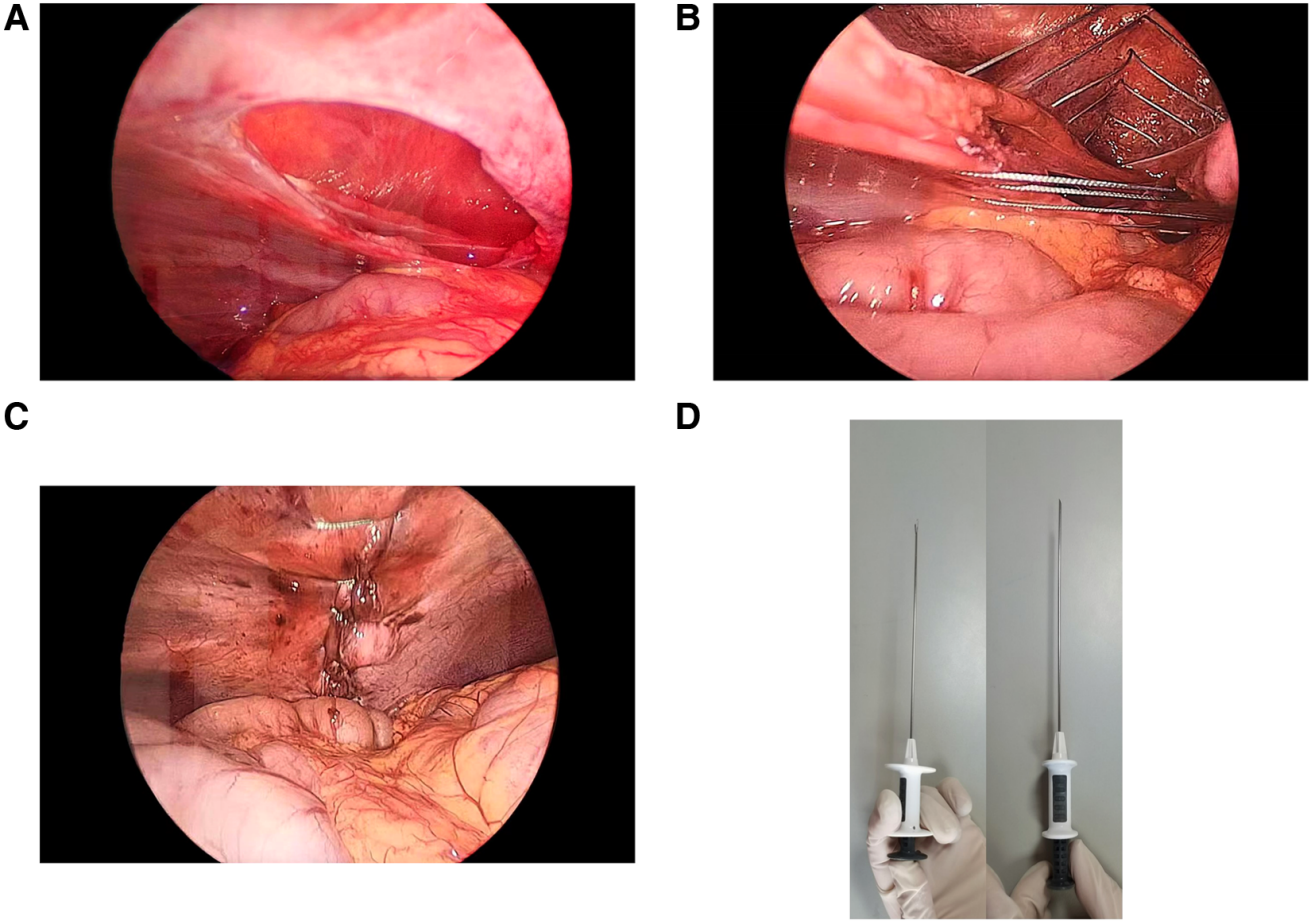
(**A**) Abdominal wall defect observed during surgery. (**B)** Intraoperative fascial closure device-assisted abdominal wall repair. (**C)** Condition of the abdominal wall after repair. (**D)** Fascial closure device used during surgery.

The patient underwent tracheal intubation and general anesthesia; a 5-mm trocar was inserted into the abdominal cavity from the umbilicus as an observation hole, and carbon dioxide was filled to create pneumoperitoneum with a pressure of 8 mmHg. Another 5-mm trocar was placed in the lower left abdomen as an operative hole. Laparoscopic exploration revealed a rupture of the muscle layer of the abdominal wall (including peritoneum, transverse abdominis, internal oblique abdominis, and external oblique abdominis) in the right abdomen, forming a 6-cm-long and 3.5-cm-wide tissue defect (Figure 3A). The small intestine herniated subcutaneously through the opening, with no signs of intestinal necrosis and perforation, and there were a small number of bloody ascites in the abdominal cavity; the liver and spleen were not damaged. We attempted to repair the muscular layer defect under laparoscopy, but due to high gap tension and difficulty in operation, we decided to use a fascial closure device (Figure 3D) to assist in suturing the muscle layer. We marked nine puncture points on the skin along the longitudinal axis of the hernia surface, with intervals of approximately 0.8 cm. The fascial closure device holding a 2-0 polyester non-absorbable suture was inserted into the skin and subcutaneous fat layer through the marked puncture points and into the abdominal cavity. The suture was guided to pass through the opposite edge of the fractured muscular layer, the tail of the suture line was grabbed, and it exited from the abdominal cavity without knotting temporarily. We used the same method repeatedly to insert and guide the suture line through the other marked puncture points (Figure 3B). Finally, each suture was tightened and knotted separately, and the tightness of the muscle layer suture was observed under laparoscopy (Figure 3C). The patient resumed a fluid diet after 4 h of surgery. On the first day after surgery, the patient got out of bed and moved around (Table 1). Two weeks after surgery, there were no abnormalities in color ultrasound, and there was no recurrence after 8 months of follow-up (Figure 1B).

**Table 1:** Timeline of treatment for the pediatric patient in the hospital.

Date	Process
25 September 2023	Being Admitted to the hospital
25 September 2023	Ultrasound and abdominal x-ray confirmed TAWH, and we performed emergency surgery
26 September 2023	The first day after surgery (stopped intensive care, electrocardiogram monitoring, and oxygen therapy, and the patient walked and moved around)
27 September 2023	The second day after surgery (pain significantly reduced)
28 September 2023	The third day after surgery (dressing changes showed good healing of the incision)
2 October 2023	Leaving hospital (the dressing was changed, and no abnormalities were seen. The patient was notified of the discharge)

## Discussion

A TAWH is relatively rare in clinical practice (3), mainly caused by the tearing of the abdominal muscle layer under tremendous external force, resulting in the protrusion of abdominal organs or tissues into the subepidermal layer. Excessive strength increases abdominal pressure, causing muscle and peritoneal lacerations, while the skin remains intact (4). Due to pain, surface congestion, etc., it is easily misdiagnosed as abdominal wall hematoma (5). Improvements are needed in relevant imaging examinations for accurate diagnosis. We used abdominal ultrasound and x-ray plain film for differentiation in this case. If it is difficult to distinguish, abdominal CT should be used to reduce misdiagnosis (6). After tissue prolapse into the body, there may be inflammatory edema, hernia ring contraction, and inability to retract hernia contents, which can lead to entrapment and serious complications such as intestinal necrosis, endangering the safety of the child's life (7). Once entrapment occurs, emergency surgery should be performed immediately, as surgical repair is the only treatment method.

There are various surgical methods for TAWH repair, most of which are open or hernia suture repair of the laparoscopic abdominal wall. For large or muscle layer defects that are difficult to suture, patches can be used to repair the defects and prevent the contents from dislodging again. Most traumatic abdominal hernias in children are treated with simple suture repair surgery because of the relatively small abdominal wall area (8). The open surgical approach involves a large incision and results in large scars, making it challenging to achieve esthetic results for younger children. Simple laparoscopic surgery faces particular difficulties in suturing muscle layers due to high abdominal wall tension. In this surgery, we used laparoscopic exploration with the assistance of a fascial closure device to successfully repair a large TAWH. This surgical method reduces the surgical complexity. It offers the following advantages: (1) Laparoscopy allows for a comprehensive examination of the overall abdominal cavity, which helps prevent overlooking any injuries to other organs within the abdomen in pediatric patients with abdominal trauma. (2) Using a fascial closure device with thread to enter multiple puncture points on the abdominal wall skin, suture the muscle layer, and then sequentially pull out the suture thread from the original puncture point to tie knots outside the body, ensures firm ligation, reduces surgical difficulty, and avoids using patches. (3) The traditional open surgery involves a long incision of the skin, which is more traumatic and leads to obvious postoperative pain. Compared with open surgery, laparoscopic surgery offers a smaller wound, improved esthetic appearance, less trauma, and faster postoperative recovery; also, the parents of the children undergoing this operation expressed high satisfaction with the results.

Due to the unique characteristics of pediatric patients, obtaining effective information can be challenging due to crying and lack of cooperation during physical examinations, leading to potential missed diagnoses. Therefore, we should combine imaging methods such as ultrasound to correctly diagnose a TAWH. There is no clear consensus on the timing of surgery. Some scholars believe that some TAWH cases can also be treated conservatively (9). However, if conservative treatment fails, repairing a longstanding TAWH often increases surgical difficulty due to organic adhesions or muscle atrophy (10). By reviewing the relevant literature, no reports were found on the application of fascial closure devices in the treatment of traumatic abdominal hernia in children. This case report shows that due to the low tension of the abdominal wall in young children, the success rate of repair is high, and no recurrence is observed during follow-up. However, for adults, the high tension of the abdominal wall and thick subcutaneous fat make the success rate of using this surgical method uncertain, needing further exploration.

## Data Availability

The original contributions presented in the study are included in the article/Supplementary Material, further inquiries can be directed to the corresponding author.
